# Genomic Insights Into Last-Line Antimicrobial Resistance in Multidrug-Resistant *Staphylococcus* and Vancomycin-Resistant *Enterococcus*

**DOI:** 10.3389/fmicb.2021.637656

**Published:** 2021-03-16

**Authors:** Adrianna M. Turner, Jean Y. H. Lee, Claire L. Gorrie, Benjamin P. Howden, Glen P. Carter

**Affiliations:** ^1^Department of Microbiology and Immunology, Doherty Institute, The University of Melbourne, Melbourne, VIC, Australia; ^2^Department of Infectious Diseases, Monash Health, Melbourne, VIC, Australia; ^3^Antimicrobial Reference and Research Unit, Microbiological Diagnostic Unit Public Health Laboratory, Department of Microbiology and Immunology, Doherty Institute, The University of Melbourne, Melbourne, VIC, Australia; ^4^Department of Infectious Diseases, Austin Health, Melbourne, VIC, Australia

**Keywords:** linezolid, daptomycin, vancomycin, genomics, *Enterococcus*, *Staphylococcus*

## Abstract

Multidrug-resistant *Staphylococcus* and vancomycin-resistant *Enterococcus* (VRE) are important human pathogens that are resistant to most clinical antibiotics. Treatment options are limited and often require the use of ‘last-line’ antimicrobials such as linezolid, daptomycin, and in the case of *Staphylococcus*, also vancomycin. The emergence of resistance to these last-line antimicrobial agents is therefore of considerable clinical concern. This mini-review provides an overview of resistance to last-line antimicrobial agents in *Staphylococcus* and VRE, with a particular focus on how genomics has provided critical insights into the emergence of resistant clones, the molecular mechanisms of resistance, and the importance of mobile genetic elements in the global spread of resistance to linezolid.

## Introduction

*Staphylococcus* and *Enterococcus* are Gram-positive cocci that are recognized as globally important opportunistic pathogens that can cause serious infections in humans, especially in hospitalized patients (Otto, 2009; Gilmore et al., 2013; Lee A.S. et al., 2018). Over recent decades, there has been a significant increase in the rates of acquired antimicrobial resistance (AMR) in these species, either through the acquisition of resistance determinants by horizontal gene transfer of mobile genetic elements (MGEs), or through mutations that alter gene expression or binding sites in native genes. This has resulted in the emergence of polyclonal lineages that are resistant to front-line therapeutic agents. In staphylococci, this includes the development and global spread of methicillin-resistant *Staphylococcus aureus* (MRSA) (Enright et al., 2002) and the recent emergence of multidrug-resistant *Staphylococcus epidermidis* (MDRSE) (Lee J.Y.H. et al., 2018). In enterococci, the emergence and dissemination of vancomycin-resistant enterococci (VRE) (Arias and Murray, 2012), particularly in two healthcare-associated species, vancomycin-resistant *Enterococcus faecalis* (VREfs) and vancomycin-resistant *Enterococcus faecium* (VREfm), is of particular clinical concern.

As resistance to different antimicrobials increases in MRSA, MDRSE, and VRE, effective and appropriate treatment becomes increasingly difficult. Limited “last-line” therapeutic options, such as linezolid, daptomycin, and, in the case of staphylococci, vancomycin, remain to treat these infections. However, the high prevalence of these species in healthcare settings (Weiner et al., 2016; Weiner-Lastinger et al., 2020), the increasing clinical use of these last-resort antimicrobials, and the ability of these species to readily develop resistance, provides strong selective pressure for the emergence of clones that are resistant to these agents. Accordingly, resistance to these last-line agents with resultant treatment failure has been increasingly reported. Individual and multiple resistance to linezolid, daptomycin, and vancomycin have been reported in clinical MRSA and MDRSE. While combined resistance to linezolid and daptomycin has recently been reported in VREfm (Wardenburg et al., 2019).

Genomic data can be used to understand the evolution of resistance and investigate the underlying resistance mechanisms. Phylogenetic analyses based on whole-genome sequencing (WGS) can also identify the presence of clones associated with specific resistance determinants, and track their emergence and global spread. The utilization of WGS in hospital infection control to identify outbreaks and transmission events associated with resistant strains is increasingly common. In this mini-review we will provide an overview of the application of genomic analyses to provide critical insights into last-line AMR in MRSA, MDRSE, and VRE, with a particular focus on the emergence of resistant clones, the molecular mechanisms of resistance, and the importance of MGEs in the global spread of linezolid resistance.

## Linezolid Resistance

**FIGURE 1:**
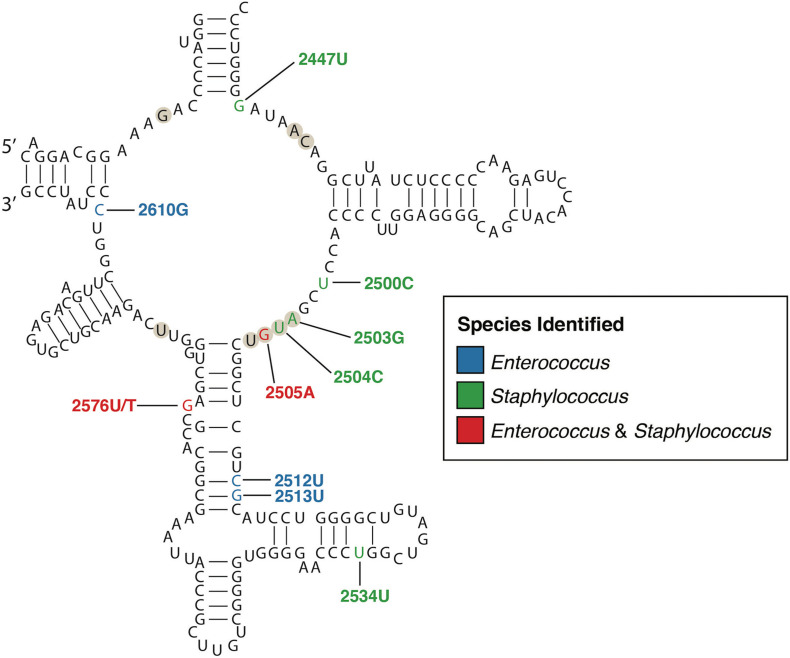
Secondary structure of the peptidyl transferase loop of domain V of 23S rRNA (*E. coli* numbering). The nucleotides that form the linezolid binding pocket are marked with circles. Nucleotide positions where mutations confer linezolid resistance are colored according to the species identified, blue for *Enterococcus*, green for *Staphylococcus*, and red for both. Only mutations with a published relationship in clinical isolates have been included (Wong et al., 2010; Long and Vester, 2012; Mendes et al., 2012; Chen et al., 2018; Wardenburg et al., 2019).

Linezolid was the first oxazolidinone approved for clinical use due to its bacteriostatic activity against Gram-positive species. Its mechanism of action inhibits protein synthesis by preventing the formation of the ternary complex between tRNA^fMet^, mRNA, and the ribosome (Hashemian et al., 2018). Although preliminary research indicated that resistance to linezolid should be rare, resistance in clinical enterococci and staphylococci have been increasingly reported since 2001 (Zurenko et al., 1996; Xiong et al., 2000; Gu et al., 2013; Bender et al., 2018a; Kosecka-Strojek et al., 2020). Early studies using PCR amplicon sequencing in clinical VRE and *S. aureus* isolates identified mutations in the 23S rRNA genes, that form the binding pocket of the ribosomal peptidyl transferase centre (PTC) to which linezolid binds (Howe et al., 2003; Meka et al., 2004; Livermore et al., 2007). A number of resistance-associated mutations, some specific to staphylococci, others conserved across staphylococci and enterococci, have been identified within and outside the PTC; the G2576T (*E. coli* nucleotide numbering) mutation is most common (Figure 1). Conferring high-level resistance in MRSA, MDRSE, and VRE, the G2576T 23S rRNA mutation is associated with linezolid treatment failure. Additional single nucleotide polymorphisms (SNPs) in the ribosomal proteins L3 and L4 have been identified through WGS of linezolid-resistant clinical staphylococci (Locke et al., 2009a,b, 2010; Endimiani et al., 2011). Mutations in the L3 and L4 proteins appear to be less common in enterococci, but several reports have identified SNPs in L3 (S133L) and L4 (T35A, N79D, I98V, and N130K) (Mendes et al., 2014; Hua et al., 2019). Further, mutations (A138G, C141T, and G166A) in ribosomal protein L22 have also been identified in linezolid-resistant staphylococci (Bender et al., 2015), as well as a S77T mutation identified in one linezolid-intermediate *E. faecium* (Lee et al., 2017). The L3, L4, and L22 proteins are in close proximity to the linezolid binding site in the ribosomal PTC, with identified mutations typically adjacent to the PTC, which may affect linezolid binding (Long and Vester, 2012). Using WGS, several studies have documented the local emergence of linezolid resistance in response to linezolid treatment during individual infections (Seedat et al., 2006; Wong et al., 2010; Chen et al., 2018). These studies identified common mutations in 23S rRNA and/or L3, L4, or L22 proteins that independently and repeatedly arise in different species of staphylococci and enterococci from different patients, demonstrating the conserved nature of mutational linezolid resistance and suggesting that convergent evolution is occurring.

### Identification of Transferable Linezolid Resistance

Transferable or acquired resistance to linezolid has been identified in both staphylococcal and enterococcal clinical isolates. The first transferable resistance gene identified was the multi-resistance gene *cfr*, that encodes a rRNA methyltransferase (Arias et al., 2008). Cfr catalyzes the post-transcriptional methylation of nucleotide A2503 in the 23S rRNA, which confers combined resistance to five different classes of antimicrobials: phenicols, lincosamides, oxazolidinones, pleuromutilins, and Streptogramin A; that bind at overlapping non-identical sites at the PTC, known as the PhLOPS_A_ phenotype (Long et al., 2006). The first linezolid-resistant clinical isolate bearing *cfr* was reported in 2005, in which *cfr* had chromosomally integrated into a MRSA strain, within an IS21-558 MGE together with *ermB*, resulting in resistance to all clinically relevant antibiotics that inhibit protein synthesis (Toh et al., 2007). The presence of a *cfr* gene has subsequently been identified in staphylococci, including MRSA (Locke et al., 2010; Morales et al., 2010; Antonelli et al., 2016) and MDRSE (Bonilla et al., 2010; Lazaris et al., 2017), and in *Enterococcus* (Liu et al., 2014; Bender et al., 2016; Fioriti et al., 2020; Ruiz-Ripa et al., 2020) isolates worldwide, although the overall prevalence and distribution in different sequence types (STs) is poorly characterized. In MDRSE, phylogenetic analysis suggests that *cfr* carriage is most commonly associated with healthcare-associated, clonal complex 2 strains (ST22, ST2, ST5, and ST168), where carriage is associated with a multidrug-resistant phenotype (Mendes et al., 2012).

Several recent studies have identified novel variants of the *cfr* gene [*cfr*(B) (Bender et al., 2016), *cfr*(C) (Candela et al., 2017), and *cfr*(D) (Guerin et al., 2020)], which are present within a variety of MGEs including multiple plasmids, insertion sequences, and transposons (summarized in Sadowy, 2018 for enterococci). Furthermore, a diverse range of resistance determinants have been co-located with *cfr*, suggesting that broader antimicrobial use may co-select for the spread of these MGEs. Variation in the genes surrounding *cfr* in clinical strains suggests that *cfr*-mediated linezolid resistance has been independently acquired on multiple occasions in clinical staphylococci and enterococci isolates worldwide. Although the reservoir for *cfr* remains unknown, one study using WGS data from *cfr*-positive *E. faecium* suggests that *E. faecium* may be an important reservoir of *cfr*(D), which has clear implications for infection control (Guerin et al., 2020). Alternatively, *cfr* variants may originate from *Bacillales*, since three *cfr*-like genes have been identified (Hansen et al., 2012).

Resistance to linezolid also arises through the acquisition of the transferable ribosomal protection genes, *optrA*, and *poxtA*. OptrA and PoxtA are part of the F-lineage in the ATP-binding cassette (ABC) superfamily of proteins that are associated with AMR (Jones et al., 2009). These ABC-F proteins mediate resistance to several different classes of anti-ribosomal antimicrobials. For instance, *optrA* confers resistance to oxazolidinones and phenicols. While, *poxtA* confers resistance to phenicols, oxazolidinones, and tetracyclines. The family of ABC-F proteins have been shown to mediate resistance by displacing the antibiotic from the ribosome to provide ribosomal protection within the *Staphylococcus* genus (Sharkey et al., 2016). OptrA was first detected in 2009, on pE349 conjugative plasmids found within three linezolid-resistant *E. faecalis* clinical isolates (Wang et al., 2015). A phylogenetic analysis of 43 *optrA*-carrying enterococci revealed nine different variants of the *optrA* gene (*optrA*_WT_, *optrA*1-5, *optrA*6, *optrA*7, and *optrA*8). These variants were distributed throughout diverse genetic backgrounds of *E. faecium* and *E. faecalis*, indicating that multiple independent acquisitions of *optrA* have occurred in these species (Bender et al., 2018b). The presence of *optrA* has been subsequently observed in both vancomycin-resistant and vancomycin-susceptible *E. faecium* and *E. faecalis* from clinical and food-producing animal isolates globally; but only reported in food-producing animal isolates of *S. aureus* (Zhu et al., 2020). This suggests that food-producing animals may serve as a reservoir for *optrA* transfer. Phylogenetic analysis of *optrA*-carrying enterococci, including some strains from a hospital outbreak, in Europe and South America led to the frequent identification of ST480 *E. faecalis*, suggesting a potential association between ST480 *E. faecalis* strains and *optrA* carriage (Sassi et al., 2019; Egan et al., 2020b; Freitas et al., 2020a; Moure et al., 2020). OptrA has also been reported in several different clonal backgrounds of *E. faecalis* (Bender et al., 2019) and has been identified in pets carrying *E. faecalis* (Wu et al., 2019). In *E. faecium*, *optrA* has been identified in several different lineages, including ST17, ST80, ST117, ST412, and ST650, indicating that *optrA* carriage is present in phylogenetically diverse strains in *E. faecium* (Morroni et al., 2018; Sassi et al., 2019).

The *poxtA* linezolid resistance gene was first detected in a MRSA clinical strain in 2016 (Antonelli et al., 2018). The *poxtA* gene exhibits genetic similarity (32% identity and 95% coverage) to *optrA* and possesses conserved features with other members of the ABC-F family (Antonelli et al., 2018). Carriage of the *poxtA* gene appears to occur sporadically in MRSA and VREfm clinical isolates (Papagiannitsis et al., 2019), with the gene most commonly identified in food-producing animals (Huang et al., 2017; Elghaieb et al., 2019; Hao et al., 2019; Lei et al., 2019; Fioriti et al., 2020; Freitas et al., 2020b; Na et al., 2020).

### Phylogenetics of Transferable Linezolid Resistance

**FIGURE 2:**
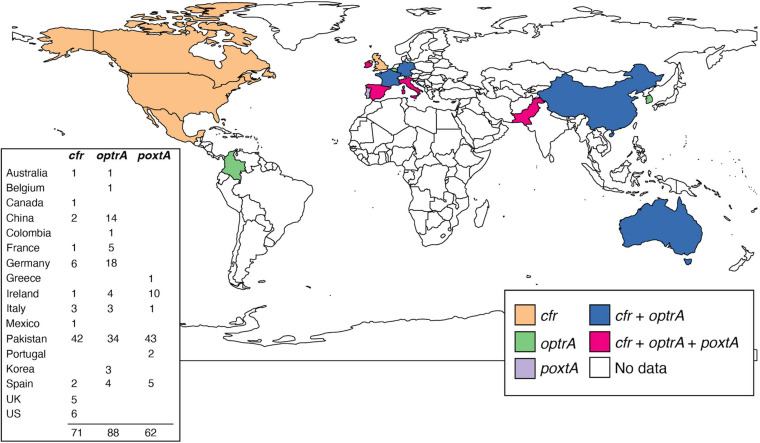
Snapshot of reported *cfr*, *optrA*, and *poxtA* genes in human, clinical, linezolid-resistant *E. faecium*. Each country is colored based on which gene(s) have been identified. The number of reported strains for each country is shown. It should be noted that the identification of each gene within regions may not accurately represent the genomic epidemiology in *E. faecium*, due to both collection and availability biases. Only published reports have been included (Bender et al., 2016, 2018b; Lazaris et al., 2017; Wardenburg et al., 2019; Egan et al., 2020a,b; Moure et al., 2020). Map was generated in R (v. 4.0.3) using the ggplot2 package (v. 1.3.0).

Since their initial discovery, the prevalence of *cfr*, *optrA*, and *poxtA* genes in clinical multidrug-resistant enterococcal and staphylococcal isolates has increased (Figure 2; Bi et al., 2018). To date, the highest prevalence of *optrA* and/or *poxtA* (35/154; 23%) were reported in *E. faecium* and *E. faecalis* in a retrospective Irish study from 2016 to 2019, suggesting significant selection pressure for gene maintenance (Egan et al., 2020b). Genomics has provided critical insights into the genetic relatedness of resistant strains, the structure of MGEs and their role in the mobilization of linezolid resistance determinants. Examples include: an analysis of *optrA*-carrying plasmids from *E. faecalis*, which found *optrA* flanked by two active copies of IS*1216* that can facilitate its spread (Shang et al., 2019); the widespread identification of *optrA* located in a Tn*6674*-like element in *E. faecalis* isolated from different countries and sources (Freitas et al., 2020b); IS*1216E* elements that have been shown to mediate the insertion of *poxtA* into a pT-E1077-31 plasmid in *E. faecium* (Shan et al., 2020); while in MRSA, a Tn*6349* composite transposon carrying both *poxtA* and *cfr* was found to possess a modular structure consisting of fragments from other previously described MGEs (D’Andrea et al., 2019). The presence of these MGEs in *cfr*, *poxtA*, and *optrA* carrying multi-resistance plasmids likely aids in the persistence and dissemination of linezolid resistance genes among these Gram-positive pathogens.

Recent studies have also used genomics to identify linezolid-resistant strains for surveillance and infection control purposes. These studies utilized different bioinformatic tools, including LRE-finder (Hasman et al., 2019) and the Resistance Gene Identifier using the Comprehensive Antibiotic Resistance Database (Alcock et al., 2020), to detect *cfr*, *optrA*, and *poxtA* resistance genes in WGS data, enabling rapid genotypic surveillance (Wardenburg et al., 2019; Eisenberger et al., 2020; Moure et al., 2020) and phylogenetic inference (Kerschner et al., 2019). For instance, WGS of *E. faecium* during an Irish hospital outbreak identified 19 nearly identical ST80 clinical isolates (allelic difference range < 10 SNPs) all carrying *optrA*-encoded within conjugative plasmid pEfmO_03. Subsequent implementation of enhanced infection control interventions successfully eliminated the outbreak (Egan et al., 2020a).

## Genomic Insights Into Daptomycin Resistance

Shortly after FDA approval of daptomycin for use in MRSA and in the context of off-label use in VRE, resistance, and corresponding clinical treatment failures were reported in both species (Skiest, 2006; Arias et al., 2007; Boyle-Vavra et al., 2011). The development of daptomycin resistance commonly coincides with prior patient exposure, indicating that therapeutic use of daptomycin is likely the main driver of resistance. In *S. aureus*, the emergence of daptomycin resistance has also been associated with the use of un-related antimicrobials, including vancomycin (Tran et al., 2015) and rifampicin (Guérillot et al., 2018b). Importantly, horizontally acquired and transferable resistance to daptomycin has not been reported to date. The worldwide prevalence of daptomycin resistance is poorly defined since few large-scale surveillance studies exist. A recent meta-analysis in *S. aureus*, suggested that only 0.1% of *S. aureus* and 0.1% of MRSA were daptomycin-resistant (Shariati et al., 2020). In enterococci, the prevalence of daptomycin resistance was estimated to be 2.6% in VREfm and 0.1% in VREfs, based on a systemic review of previous studies (Kelesidis et al., 2011). Little is known about which lineages of staphylococci and enterococci are associated with the development daptomycin resistance. In *S. aureus*, genomic analyses indicate that daptomycin resistance has arisen in ST5, ST398, and ST22 isolates, based on data from the United States and South America (Dabul and Camargo, 2014; Damasco et al., 2019). In *S. epidermidis*, no comprehensive phylogenetic analyses of daptomycin-resistant strains have been completed, but resistance has been reported in ST2, internationally (Lee J.Y.H. et al., 2018). Similarly, no phylogenetic studies have been completed for *E. faecalis*, but a novel ST736 clone associated with daptomycin resistance has been identified for *E. faecium* (Wang et al., 2014) in some United States hospitals (Wang et al., 2018), as has daptomycin-resistant ST80 *E. faecium* (Udaondo et al., 2020).

Daptomycin’s mode of action involves binding to the anionic phosphoglycerol and various undecaprenyl-coupled cell envelope precursors within the bacterial cell membrane in the presence of Ca^2+^, blocking cell wall synthesis and triggering delocalization of biosynthetic proteins from the membrane, resulting in bacterial cell death (Grein et al., 2020). Although the specific mechanisms leading to resistance are not fully understood, comparative genomic studies have been pivotal in identifying genetic determinants of daptomycin resistance. Genetically, a critical step toward developing a resistant phenotype appears to involve mutations in two groups of genes: (1) bacterial regulatory systems that function in cell wall homeostasis and (2) enzymes involved in membrane phospholipid metabolism.

In *S*. *aureus* and *S. epidermidis*, mutations in *mprF* (S295L, S337L, T345L, I420N, and L826F) and *walKR* (*yycFG*) (WalK^V500F^ and WalR^R216S^) are associated with the development of daptomycin resistance in clinical isolates and laboratory-derived strains (Howden et al., 2011; Yang et al., 2009, 2013; Jiang et al., 2019). The MprF enzyme produces cationic phospholipid lysylphophatidyl-glyercol (LysPG) and flips it from the inner to outer leaflet of the cytoplasmic membrane, which decreases the negative cell surface charge (Oku et al., 2004). Although not characterized in *S. epidermidis*, the mutations in *S. aureus* likely increase the enzymatic activity of MprF since daptomycin-resistant isolates display increased levels of LysPG in their membrane, which variably leads to a more cationic cell surface that electrostatically repels the daptomycin-Ca^2+^ complex (Mishra et al., 2011). For mutations in *walKR*, the mechanism leading to resistance is distinct in *S. aureus* and *S. epidermidis*, since *S. aureus* strains carrying mutations in *walKR* display increased surface charge while *S. epidermidis* strains display decreased biofilm formation, compared to the wild-type strains (Jiang et al., 2019). Comparative genomic studies in *S. aureus* have also identified mutations in the cardiolipin synthase gene *cls*2 and RNA polymerase β and β’ subunits (*rpoB* and *rpoC*) that are associated with resistance to daptomycin, although these are less frequently observed than mutations in *mprF* and *walKR* (Miller et al., 2016). Transposon-sequencing in *S. aureus* has also identified multi-component sensing genes, specifically *graRS* and *vraFG* (which regulate cell envelope processes), as being associated with daptomycin resistance, with these genes being important for survival following daptomycin stress (Rajagopal et al., 2016; Coe et al., 2019). However, *graRS* and *vraFG* mutations have so far not been described in clinical daptomycin-resistant *S. aureus* isolates.

In enterococci, comparative genomic studies have shown that mutations in the three-component regulatory system *liaFSR* and cardiolipin synthase *cls* are associated with the development of clinical daptomycin resistance. Mutations within the LiaFSR three-component system, which regulates the cell envelope response to cationic host defense peptides and antimicrobial stress, have been frequently identified in daptomycin-resistant clinical isolates, with the LiaS^T120A^ and LiaR^W73C^ mutations being of particular importance (Arias et al., 2011). A genomic analysis of *E. faecium* clinical isolates found that isolates with higher daptomycin MICs (≥ 4 mg/L) usually contained mutations in the *liaFSR* system (Diaz et al., 2014). Mutations in Cls (H215R and R218Q), encoding a transmembrane protein that catalyzes the synthesis of cardiolipin from phosphoglycerol, are also frequently associated with daptomycin resistance (Arias et al., 2011; Palmer et al., 2011). However, molecular studies have thus far not demonstrated a causal relationship between *cls* and daptomycin resistance in *E. faecium* (Tran et al., 2013). Since *cls* mutations are often found in conjunction with substitutions in the *liaFSR* regulon, they likely contribute to the progression of an isolate developing a daptomycin-resistant phenotype rather than independently causing resistance (Davlieva et al., 2013). Recent genomic studies in enterococci have also identified daptomycin-resistant isolates that contain wild-type *liaFSR* and *cls* alleles, indicating that other unknown genetic determinants of daptomycin resistance exist that require further investigation (Wang et al., 2018; Prater et al., 2019). Indeed, putative associations with daptomycin resistance and mutations within *mprF, cfa, pgsA*, and *dlt* have been suggested, but have not yet been verified.

## Genomic Insights Into Vancomycin Resistance in *Staphylococcus*

Vancomycin is a critical last-line antibiotic for treating infections caused by multidrug-resistant *Staphylococcus*. Thirty-nine years after FDA approval of vancomycin, the first instances of *S. aureus* with intermediate vancomycin resistance (MICs 4 to 8 mg/L) were reported (Sieradzki et al., 1999; Smith et al., 1999). Soon after, the first clinical case of a vancomycin-resistant *S. aureus* isolate (MIC 1024 mg/L), carrying the *vanA* vancomycin resistance operon, was reported (Chang et al., 2003). Subsequent molecular studies demonstrated that the *vanA* operon originated from a co-infecting VREfs strain, raising significant concerns about the potential dissemination of vancomycin resistance in *S. aureus* from VRE reservoirs (de Niederhäusern et al., 2011). Fortunately, this has not eventuated. Detailed genomic studies have demonstrated that the transfer of the *van* operon to *S. aureus* rarely occurs, with very few reports worldwide (McGuinness et al., 2017). The rare occurrence of VRSA may be due to the fitness cost associated with carriage of the *van* genes, as a longer lag phase was observed during *in vitro* growth with vancomycin, and the plasmid carrying the *van* genes was found to be genetically unstable. Further, a MRSA PBP2a strain was unable to utilize lipid II with the D-Alanyl-D-lactate alteration conferred by the *van* operon (Foster, 2017). A single study identified *S. aureus* clonal complex 5 (CC5) as the genetic background in which the *van* operon is most commonly acquired. This study found that CC5 vancomycin-resistant *S. aureus* (VRSA) strains were genetically distinct, indicating independent acquisition of the *vanA* operon had occurred (Kos et al., 2012). This suggests that the spread of *van* operons within *S. aureus* may occur should appropriate selective conditions develop. Continued vigilance is therefore paramount.

Vancomycin-intermediate *S. aureus* (VISA) resulting from the progressive accumulation of mutations in key essential and regulatory genes are clinically more common than true vancomycin-resistant *S. aureus*. VISA isolates are commonly characterized by a thickened cell wall, slower growth, and increased autolysis (reviewed in Howden et al., 2010). Genetically, VISA strains typically contain mutations in two-component regulators *vraRS* (Cui et al., 2009), *graRS* (Cui et al., 2009), and *walKR* (Howden et al., 2011), which function in cell-wall synthesis, but also may contain mutations in the rifampicin resistance determining region of *rpoB* (Cui et al., 2010). A convergent evolution analysis of 7,099 *S. aureus* genomes suggested that clinical rifampicin use may promote the emergence of multidrug-resistant lineages of *S. aureus* that contain rifampicin resistance mutations in *rpoB* that also confer intermediate resistance to vancomycin (Guérillot et al., 2018a). Although VISA can be found in any genetic background, ST5 is most commonly associated with VISA (Howden et al., 2014), although ST239 isolates tend to display the highest vancomycin MICs (Holmes et al., 2014). Genome-wide association studies (GWAS) performed in ST239 *S. aureus* have identified mutations in *rpoB* (H481Y/L/N) (Alam et al., 2014) and the *walKR* genes (Baines et al., 2015) as being most strongly associated with the development of the VISA phenotype.

In *S. epidermidis*, the global dissemination of three nearly pan-drug-resistant lineages (two ST2 and one ST23) with heteroresistance to vancomycin was recently demonstrated (Lee J.Y.H. et al., 2018). Analyses of WGS data from 419 clinical isolates from 96 institutions in 24 countries, sampling at least 77 ST types, identified dual D471E and I527M RpoB mutations to be the most common cause of rifampicin resistance in *S. epidermidis*, accounting for 86.6% of mutations. Of note, the D471E and I527M combination occurred almost exclusively in isolates from the ST2 and ST23 lineages. Through site-specific mutagenesis, the dual RpoB mutation was shown to confer high-level rifampicin resistance as well as reduced susceptibility to vancomycin and teicoplanin. The presence of these same mutations in multiple genetically diverse backgrounds was consistent with their independent emergence with subsequent fixation, presumably due to their advantageous antimicrobial resistant phenotype. Furthermore, acquisition of additional resistance to linezolid (mediated by *cfr* carrying plasmids and/or mutations in 23S rRNA, L3 and L4) and daptomycin (substitutions in *mprF*) rendered some European *S. epidermidis* essentially untreatable. Further examples of convergent evolution include the presence of H481Y/L/N substitutions in RpoB that confer vancomycin resistance in both *S. aureus* (Guérillot et al., 2018a) and *S. epidermidis* (Gao et al., 2013; Lee J.Y.H. et al., 2018; Dao et al., 2020).

## Conclusion

Collectively, MRSA, MDRSE, and VRE pose a significant clinical and economic healthcare burden. This has been compounded by increasing resistance to the key last-line agents daptomycin, linezolid and in the case of *Staphylococcus*, vancomycin. Genomic analyses have been pivotal in providing evolutionary insights into the underlying genetic mechanisms of resistance that have arisen in all three species. In particular, the identification of transferable and acquired linezolid resistance determinants through genomic studies, has been critical for understanding the global dissemination and genetic mobility of these genes. Genomic analyses using WGS data is therefore a powerful method for understanding last-line AMR in these and other clinically relevant multidrug-resistant species.

## Author Contributions

GC and AT conceptualized the mini-review topic. All authors contributed to writing the mini-review and editing the final version.

## Conflict of Interest

The authors declare that the research was conducted in the absence of any commercial or financial relationships that could be construed as a potential conflict of interest.
